# Determination of DNA recovery from human teeth exposed to various acids

**DOI:** 10.1007/s00414-025-03445-x

**Published:** 2025-02-18

**Authors:** Tuğba Ünsal Sapan, Nejla Karaboğa

**Affiliations:** 1 Institute of Addiction and Forensic Sciences, Üsküdar University, E Block, Central Campus, İstanbul, 34662 Türkiye; 2 Faculty of Engineering and Natural Sciences, Department of Forensic Sciences, Üsküdar University, İstanbul, 34662 Türkiye

**Keywords:** Forensic sciences, Forensic genetics, Genetic identification from dental samples, Acid exposure, Hydrochloric acid (HCl), Nitric acid (HNO_3_), Sulfuric acid (H_2_SO_4_)

## Abstract

Forensic sciences encompass a comprehensive application of various scientific disciplines working together to solve crimes. In forensic cases, identification is the primary procedure. Due to the variety of crimes, difficulties can arise in identification. The ability to extract DNA from all types of biological tissues has made DNA analysis one of the most frequently used and reliable methods in identification. Teeth, which are noted for their relatively low degradation over long periods despite exposure to environmental, physical, and chemical factors, and their ability to securely preserve DNA material, are among the most used samples in DNA analysis. With the evolution of crime and methods of concealing it, body integrity-threatening procedures such as exposure to acids are frequently used by perpetrators. However, several studies have shown that DNA can still be recovered from dental samples exposed to various chemicals for specific durations. This study aimed to recover DNA from teeth exposed to strong acids. Dental samples obtained from volunteers were exposed to strong acids, including HCl, HNO_3_, and H_2_SO_4_, for 8, 12, and 120 hours, respectively, with each duration parameter tested separately. Following exposure, DNA isolation was performed, and the amount of DNA was assessed. Samples deemed suitable for identification underwent PCR and electrophoresis to obtain DNA profiles. The results showed that it was possible to successfully obtain a full DNA profile from molars exposed to sulfuric, hydrochloric, and nitric acid for up to 24 h, while it was possible to obtain a partial DNA profile from molars exposed to sulfuric acid for up to 120 h.

**Supplementary Information:**

The online version contains supplementary material available at 10.1007/s00414-025-03445-x.

## Introduction

Various methods are employed for the identification of human remains, depending on the conditions in which the remains are found and the duration of exposure to these conditions. In forensic cases, methods that are easy and quick are primarily preferred. While these methods may involve identification by relatives or family members of the deceased, identification becomes impossible in cases where the body has severely decomposed, resulting in the disruption of body integrity. Therefore, interdisciplinary identification studies involving forensic sciences are conducted [1, 2].

Teeth are a crucial part of the human skeleton. Among all bones, teeth are the structures with the greatest chemical and physical durability and the most protection. Unlike other skeletal bones, the crown of the teeth is only affected by trauma, wear, treatments, and mineralization disorders. Teeth contain a wealth of information about an individual, including sex, age, health status, diet, diseases, and occupation. Due to their robust structure, which preserves genetic material, teeth are frequently used in DNA analysis to obtain profiles [3, 4].

The aim of this study is to identify the challenges encountered in cases where dental samples are used for individual identification and to determine whether DNA can be recovered under these conditions. This study examines teeth, which are the most protected and robust structures that preserve DNA information, in scenarios where the body or remains are subjected to various strong acids to destroy evidence, such as in disaster victims or criminal cases. Previous studies have demonstrated that DNA can be recovered from dental samples exposed to specific acids. In this study, the goal is to recover DNA from different dental samples exposed to various strong acids: 37% Hydrochloric Acid (HCl), 65% Nitric Acid (HNO_3_), and 95–98% Sulfuric Acid (H_2_SO_4_).

## Method

### Sample preparation

All samples were collected following written informed consent, in accordance with the Declaration of Helsinki and national regulations, and with the approval of the University Non-Interventional Research Ethics Committee (approval number: 61351342/EKİM 2023-39). The study adhered strictly to ethical guidelines to ensure the rights and safety of participants were protected throughout the research process. A total of 52 tooth samples were collected from healthy volunteers over the age of 18 who provided informed consent and signed approval forms. These individuals were patients who visited the dentist due to dental issues and had teeth deemed necessary for extraction. In this study, nitric acid (65% HNO_3_), hydrochloric acid (37% HCl), and sulfuric acids (95–98% H_2_SO_4_) were employed in their highest available concentrations to achieve the most effective DNA recovery. The chosen durations, 8 h, 24 h, and 120 h were selected to assess the extent of damage and to compare the results in DNA analysis. The 8-hour duration was chosen to evaluate the effects of short-term exposure, while the 24-hour duration was selected based on the assumption that it would lead to a more significant level of DNA degradation. The 120-hour duration for sulfuric acid was set to induce the targeted morphological changes. Reference samples were used for comparison with all other samples.

Sixteen dental samples were exposed to nitric acid (HNO_3_) in two groups: one for 8 h and one for 24 h. Sixteen dental samples were exposed to hydrochloric acid (HCl) in two groups: one for 8 h and one for 24 h. Eighteen dental samples were exposed to sulfuric acid (H_2_SO_4_) in three groups: 8 h, 24 h, and 120 h.

One incisor and one molar tooth were used as reference samples and were not exposed to any acids, proceeding directly to the cleaning stage.

After acid exposure, the dental samples were placed in a 5% sodium hypochlorite solution for 20 min using sterile forceps. The samples were then transferred to a drying paper using sterile forceps. Mechanical cleaning was performed to remove excess tissue from the outer surface using a sterile paper file. After the sanding process, the samples were cleaned sequentially in 100% ethanol and distilled water within a funnel. After all cleaning procedures, the samples were left to dry on the paper to be prepared for pulverization.

To isolate dried tooth samples, the QIAamp^®^ DNA Investigator Kit (Qiagen) was utilized following the standard protocol for bone and tooth samples. The tooth samples were first pulverized using nitrogen and a metal blender in accordance with the specified procedure. Each tooth sample was carefully transferred from the blotting paper to a steel container, which was sterilized along with the metal blender used for the shredding process.

Wearing protective gloves, liquid nitrogen was introduced into the steel container containing the tooth sample from the liquid nitrogen tank. A metal blender was then placed on top of the sample, which was subsequently wrapped in foil. The metal blender was operated until the tooth samples were sufficiently pulverized. The pulverized tooth samples were then weighed to less than 100 mg, as required by the protocol.

### DNA extraction and quantification

The dried dental samples were isolated using the QIAamp^®^ DNA Investigator Kit following the Bone and Teeth procedure. For teeth dissolved in acid, isolation was performed using the QIAamp^®^ DNA Investigator Kit Body Fluid Stains procedure. The amount of DNA in the samples was measured using the Qubit 4.0 Fluorometer and kit The Qubit 1X dsDNA HS Assay Kit [5].

### PCR and electrophoresis

For the PCR, the GlobalFiler™ PCR Amplification Kit (ThermoFisher Scientific) was used, and procedures were conducted according to the kit instructions. For each sample, 7.5 µl of Master Mix, 2.5 µl of Primer Set, and 15 µl of DNA sample were combined to prepare a total of 25 µl PCR mixtures, applied to three samples. The prepared PCR mixtures were placed in the Veriti™ 96-Well Thermal Cycler and run according to the cycle conditions recommended for the GlobalFiler™ PCR Amplification Kit. The number of cycles under PCR conditions was modified to 34 cycles due to the presence of trace samples. The PCR process initially involved heating at 95 °C for 1 min, followed by 34 cycles of 94 °C for 10 s each. Subsequently, 34 cycles at 59 °C for 90 s each were performed. The PCR cycle concluded with a 10-minute step at 60 °C, followed by storage at 4 °C.

The PCR products were then prepared for the electrophoresis stage. Considering negative and positive controls along with the allele ladder, a 10 µL mixture of Hi-Di™ Formamide and 0.5 µL of GeneScan™ 600 LIZ™ Size Standard v2.0 was prepared and vortexed for each sample. This mixture was added as 10 µL to each plate well, followed by the addition of 1 µL of the PCR product. The amplified PCR products were sequentially placed into the 3500 Genetic Analyzer. The resulting data were analyzed using GeneMapper™ ID-X Software [6].

## Results

### Nitric acid exposed samples

**Fig. 1:**
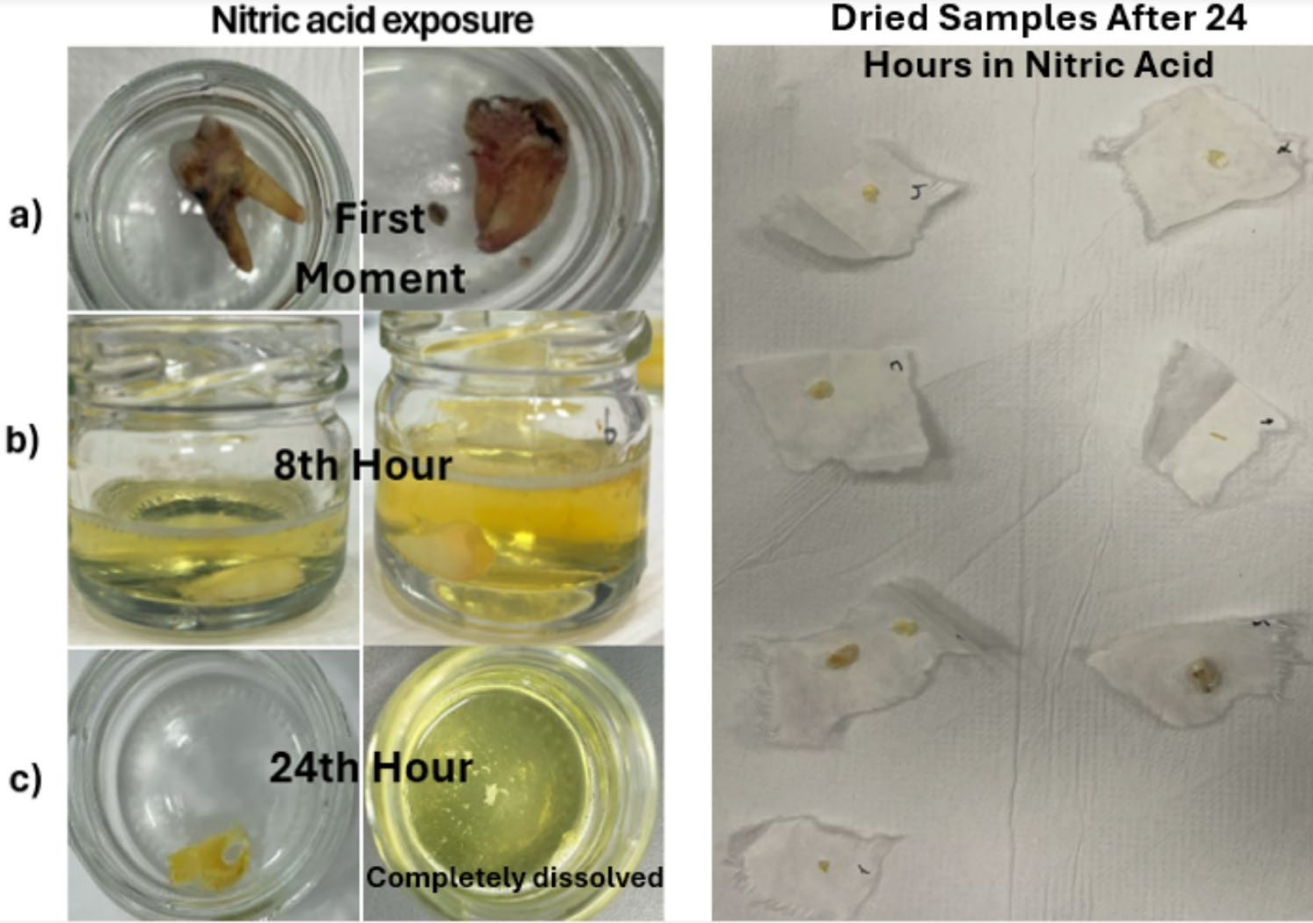
Images of dental specimens after nitric acid exposure. * (**a**) First moment, (**b**) After 8 h of Nitric Acid Exposure, (**c**) After 24 h of Nitric Acid Exposure

In dental samples exposed to nitric acid for 8 h, complete dissolution was not observed. A reduction in the outer layer of the tooth structure was noted. The color of the acidic solution was observed to be yellow. It was evident that blood and tissue residues on the outer surface of the tooth samples dissolved in the nitric acid (Fig. 1. (b)). No significant differences were observed in the colors of the acidic solutions containing the samples.

Teeth exposed to nitric acid for 24 h underwent significant morphological changes. In this group, one tooth sample became liquefied. Upon removal of the teeth, the blood and tissue residues on their surfaces were completely dissolved in the nitric acid and were not observed in the final analysis. After 24 h, the color of the liquid in the glass container holding the samples was yellow (Fig. 1. (c)). When the tooth was completely dissolved, the liquid appeared a lighter shade of yellow.

### Hydrochloric acid exposed samples

**Fig. 2:**
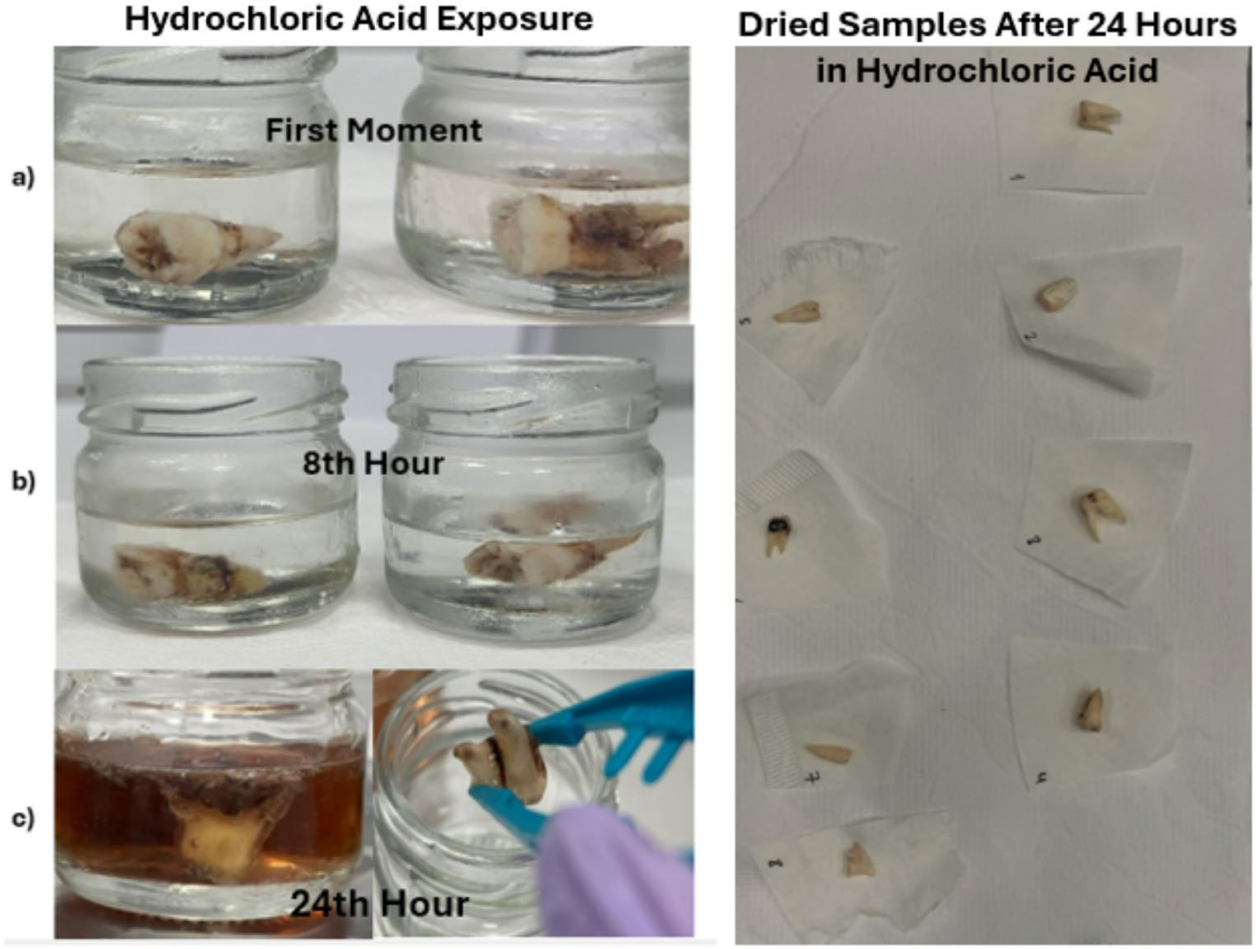
Images of dental specimens after hydrochloric acid exposure. *(**a**) First moment, (**b**) After 8 h of Hydrochloric Acid Exposure, (**c**) After 24 h of Hydrochloric Acid Exposure

In dental samples exposed to hydrochloric acid for 8 h, complete dissolution was not observed. Blood and tissue residues on the outer surfaces of the teeth continued to persist in the hydrochloric acid solution. (Fig. 2(b)) The color of the acidic solution varies, with darker colors observed in samples with higher amounts of blood and tissue on the tooth surfaces, and lighter colors in samples with less residual material.

Teeth exposed to hydrochloric acid for 24 h underwent significant morphological changes. In this group, one tooth sample had liquefied. Despite this, blood, and tissue residues on the surfaces of the teeth did not disappear. In some samples, residue remained attached to the teeth, while in others, they were present separately in the acidic solution (Fig. 2 (c)). The color of the acidic solution appeared lighter in samples closer to complete dissolution of the tooth. The outer surfaces of the teeth displayed a slightly gelatinous and translucent texture, showing a slick appearance.

### Sulfuric acid exposed samples

**Fig. 3:**
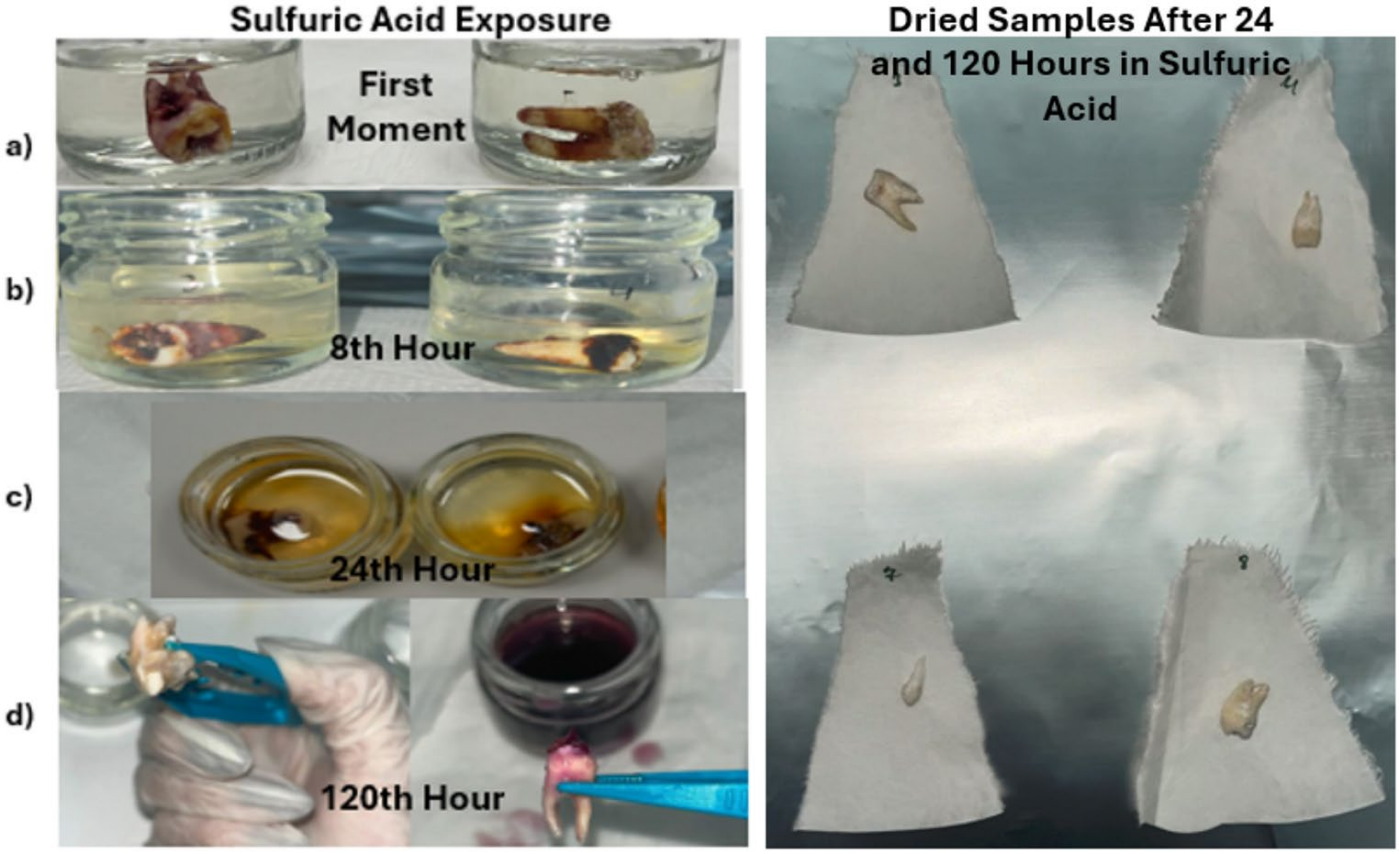
Images of dental specimens after sulfuric acid exposure. *(**a**) First Moment, (**c**) After 8 h of Sulfuric Acid Exposure, (**c**) After 24 h of Sulfuric Acid Exposure, (**d**) After 120 h of Sulfuric Acid Exposure

In samples exposed to sulfuric acid for 8 h, no morphological changes were observed. Blood and tissue residues on the outer surfaces of the teeth continued to persist and were not dissolved. Minor differences in the color of the acidic solutions were noted compared to their initial state. However, these color changes did not significantly affect the color or clarity of the solution. (Fig. 3 (b)) During the cleaning stage with sodium hypochlorite, it was observed that a white tissue formation mostly occurred on the crown (coronal) area of the dental samples.

Teeth exposed to sulfuric acid for 24 h did not exhibit significant morphological changes. The blood and tissue residues on the surfaces of the teeth continued to persist. The color of the sample solution darkened due to the presence of blood and tissue residues. (Fig. 3 (c)) A creamy, dissolved tissue formation was observed on the outer surfaces of the teeth.

In samples exposed to sulfuric acid for 120 h, creamy tissue formations were observed on the outer surfaces. The acidic solution appeared dark, and its contents were difficult to discern. The tissue and blood residues of the teeth were completely separated and dissolved in the acidic solution. (Fig. 3 (d)).

### DNA quantification

In this study, DNA recovery was performed on 50 dental samples in pulverized form and 2 samples in liquid form. The samples were processed using the QIAamp^®^ DNA Investigator Kit by QIAGEN, following the procedures appropriate for Bone and Teeth and Body Fluid Stains samples, with DNA isolation conducted via the spin column method. After the isolation process, the DNA quantities were measured using a Qubit 4.0 Fluorometer with the Qubit dsDNA HS Assay Kit. DNA recovery was successful in 51 out of 52 samples, except for one sample.

In samples exposed to nitric acid for 8 h, DNA quantities ranged from 0.194 ng/µl to 16.3 ng/µl. In the 24-hour group, the DNA amounts varied between 0.244 ng/µl and 0.0260 ng/µl. For the sample that had completely dissolved in nitric acid, the DNA isolation yielded a quantity of 0.0302 ng/µl. Among the samples exposed to nitric acid, those left for 8 h had an average DNA recovery of 4.339 ng/µl, while those exposed for 24 h had an average recovery of 0.113 ng/µl (Table 1).

**Table 1:** DNA quantities of samples exposed to nitric acid

Exposure to Nitric Acid							
Sample No	Tooth Type	Reference Samples (Not exposed to acid)	DNA Quantities After 8 h (ng/µl)	Average DNA Quantities After 8 h (ng/µl)	Tooth Type	DNA Quantities After 24 h (ng/µl)	Average DNA Quantities After 24 h (ng/µl)
Ref 1	Molar	7.74					
Ref 2	Incisor	1.72					
1	Molar		4.50	4.339	Molar	0.244	0.113
2	Molar		0.592		Molar	0.167	
3	Molar		4.12		Molar	0.0260	
4	Molar		0.194		Molar	0.0768	
5	Molar		16.3		Molar	0.208	
6	Molar		0.772		Incisor	0.0652	
7	Molar		2.50		Molar	0.0888	
8	Molar		5.74		Molar	0.0302	

In samples exposed to hydrochloric acid for 8 h, DNA quantities ranged from 0.0426 ng/µl to 7.92 ng/µl. In the 24-hour samples, DNA amounts varied between 0.0301 ng/µl and 0.171 ng/µl, with one sample yielding no detectable DNA. Among the samples exposed to hydrochloric acid, those left for 8 h had an average DNA recovery of 2.885 ng/µl, while those exposed for 24 h had an average recovery of 0.0817 ng/µl. From the sample that had liquefied, a DNA recovery of 0.0398 ng/µl was obtained (Table 2).

**Table 2:** DNA quantities of samples exposed to hydrochloric acid

Exposure to Hydrochloric Acid							
Sample No	Tooth Type	Reference Samples (Not exposed to acid) (ng/µl)	DNA Quantities After 8 h (ng/µl)	Average DNA Quantities After 8 h (ng/µl)	Tooth Type	DNA Quantities After 24 h (ng/µl)	Average DNA Quantities After 24 h (ng/µl)
Ref 1	Molar	7.74					
Ref 2	Incisor	1.72					
1	Molar		2.99	2.885	Molar	0.171	0.0817
2	Molar		5.18		Molar	0.154	
3	Molar		3.15		Molar	0.0324	
4	Molar		1.84		Incisor	-	
5	Molar		7.92		Incisor	0.0398	
6	Molar		1.05		Molar	0.0328	
7	Molar		0.905		Incisor	0.0534	
8	Molar		0.0426		Incisor	0.0301	

In samples exposed to sulfuric acid for 8 h, DNA quantities ranged from 0.0233 ng/µl to 7.65 ng/µl. For the 24-hour samples, DNA amounts varied between 0.0210 ng/µl and 0.255 ng/µl. In samples exposed to sulfuric acid for 120 h, DNA quantities were found to range from 0.0318 ng/µl to 0.0456 ng/µl. Among the samples exposed to sulfuric acid, those left for 8 h had an average DNA recovery of 1.870 ng/µl, those exposed for 24 h had an average recovery of 0.0828 ng/µl, and those left for 120 h had an average recovery of 0.0387 ng/µl (Table 3).

**Table 3:** DNA quantities of samples exposed to sulfuric acid

Exposure to Sulfuric Acid										
Sample No	Tooth Type	Reference Samples (Not exposed to acid) (ng/µl)	DNA Quantities After 8 h (ng/µl)	Average DNA Quantities After 8 h (ng/µl)	Tooth Type	DNA Quantities After 24 h (ng/µl)	Average DNA Quantities After 24 h (ng/µl)	Tooth Type	DNA Quantities After 120 h (ng/µl)	Average DNA Quantities After 120 h (ng/µl)
Ref 1	Molar	7.74								
Ref 2	Incisor	1.72								
1	Molar		0.0355	1.870	Molar	0.255	0.0828	Molar	0.0456	0.0387
2	Molar		2.45		Molar	0.0586		Molar	0.0318	
3	Molar		0.0233		Molar	0.0338				
4	Molar		3.06		Molar	0.0210				
5	Molar		0.0860		Molar	0.0760				
6	Molar		1.35		Incisor	0.0310				
7	Molar		0.310		Incisor	0.0274				
8	Molar		7.65		Molar	0.160				

### Identification

Identification was made from samples exposed to acid for 8, 24 and hours and containing more than 0.125 ng of DNA. For the identification process, the GlobalFiler™ PCR Amplification Kit was used. Based on the electrophoresis results, the most successful profile was obtained from the nitric acid sample. The sample treated with nitric acid for 8 h produced a complete STR profile, with alleles detected at all 24 loci, except for DYS391. In contrast, the sample exposed to nitric acid for 24 h exhibited alleles at 17 of the 24 loci. Similarly, the sample treated with hydrochloric acid for 8 h also provided a complete STR profile, with alleles detected at all 24 loci, excluding DYS391. After 24 h of hydrochloric acid exposure, Sample 1 showed alleles at 18 out of 24 loci, while Sample 2 displayed alleles at only 13 out of 24 loci. Finally, the sample exposed to sulfuric acid for 8 h yielded a full DNA profile, with alleles detected at all 24 loci, except DYS391. In contrast, after 24 h of sulfuric acid exposure, Sample 1 exhibited alleles at 20 out of 24 loci, while Sample 2 showed alleles at 12 out of 24 loci. And finally, after 120 h of sulfuric acid exposure a sample exhibited alleles at 8 out of 24 loci as partial profile (Supplementary Material, Table 4).

**Table 4:** DNA profile of tooth samples exposed to nitric acid and hydrochloric acid for 8 and 24 h

STR Loci	Exposed Nitric Acid		Exposed Hydrochloric Acid			Exposed Sulfuric Acid			
	8 h Sample (4.12 ng/µl)	24 h Sample (0.244 ng/µl)	8 h Sample (1.84 ng/µl)	24 h Sample 1 (0.171 ng/µl)	24 h Sample 2 (0.154 ng/µl)	8 h Sample (7.65 ng/µl)	24 h Sample 1 (0.255 ng/µl)	24 h Sample 2 (0.160 ng/µl)	120 h Sample (0.0456 ng/µl)
D3S1358	15/16	14/15	16/17	14/15	14/15.2	16/17	14/15	10/12	15/15
vWA	17/17	14/14	14/14	14/16	14/15	16/17	14/16	13/13	
D16S639	12/13	12/12	11/11	9/10	-	9/13	9/11	10/10	
CSF1PO	10/11.3	-	10/11	9/10	-	12/13	10/10	-	
TPOX	9/11	-	9/10	8/11	-	7.3/11		-	
Y INDEL	2	2	2	2	2	2	2	1	2
AMEL	X/Y	X/Y	X/Y	X-Y	X/Y	X/Y	X/Y	X/Y	X/Y
D8S1179	12/13	13/14	11/14	10/14	10/10	13/14	10/14	10/10	
D21S11	31/32	30/30	28/29	24.3/31	-	28/31.2	30/31.2	28/28	
D18S51	11/12	-	15/17	-	-	14/18	-	-	
DYS391	-	-	-	-	-	-	-	-	
D2S441	11/14	11/11.3	11/14	11/14	11/14	11/13.3	11/14	10/11	11/12
D19S433	12/14.3	13/13.2	13.2/18.2	12.2/13.2	13.2/13.2	11.2/14	13.2/15	13.2/13.2	12.2/13.1
TH01	7/9.3	6/7	7/9	6/7	7/7	9/9.3	6/7	7/7	-
FGA	20/21	22/24	21/25	22/24	-	22/23	22/25	22/22	-
D22S1045	15/16	15/16	14/16	15/15	14/15	11/16	14/15	-	15/16
D5S818	10/12	9/9	9/10	12/13	11/12	10/13	12/13	-	9/9
D13S317	11/11	8/11	9/14	-	11/12	9/12	11/13	-	-
D7S820	11/11	-	10/11	-	-	9/11	-	-	-
SE33	20/21		18/27.2	-	-	21/24.2	16/16	-	-
D10S1248	13/14	11/14	13/14	13/15	13/15	13/15	13/15	13/15	11/14
D1S1656	17.3/17.3	12/17.3	12/15.3	12/17	12/17	13/15	11/12	-	-
D12S391	19/20	17/17	17/17	-	-	18/19	24/25	-	-
D2S1338	18.3/23	-	20/20	17/19.3	-	17/19.3	25/25	-	-

## Discussion

Teeth represent a crucial component of the human skeleton. Among all bones, teeth are the most chemically and physically resilient and well-preserved structures. The mineral composition of teeth allows for the long-term preservation and analysis of DNA, which is a significant advantage in solving crimes that occurred a long time ago. The use of strong acids to destroy bodies is one of the methods employed by criminals. To investigate this issue, the most durable structures, namely tooth samples, were chosen. The parameters affecting the destruction of tooth samples by acids include the duration of exposure and the strength of the acids. As the duration and strength of the acid increase, the extent of dissolution also increases. Studies on teeth exposed to strong acids have concluded that DNA analysis cannot be performed conclusively when the teeth are completely dissolved [7–9].

To observe the DNA amount recoverable from teeth without exposure to any chemicals, molar and incisor teeth were selected. DNA amounts of 7.74 ng/µl were obtained from the molar, which is significantly higher compared to 1.72 ng/µl from the incisor. The analysis of both acid-exposed and reference samples demonstrated that the highest DNA recovery was achieved predominantly from molars, whereas the lowest recovery was observed in incisors. Notably, acid-exposed molars yielded DNA quantities comparable to those recovered from incisors. Despite this, the DNA recovered from molars was sufficient for identification, whereas none of the incisors provided adequate DNA for identification purposes (Tables 1 and 2, and 3). These results further highlight the superior efficiency of molars in DNA recovery.

### Nitric acid exposure

The effect of nitric acid on tooth samples increases with exposure time, leading to more effective dissolution with prolonged exposure. The color of the acidic solution and the morphological changes in the teeth reflect the dental material’s reaction to the acid. Changes in solution color indirectly indicate the degree of dissolution.

Among the DNA isolation results, the group exposed for 8 h was more successful in terms of DNA yield compared to the 24-hour group, as anticipated. For the 8-hour exposure group, DNA amounts ranging from 0.194 ng/µl to 16.3 ng/µl were obtained. In the 24-hour group, most of the tooth morphology had been lost, which suggests that the acid had largely destroyed the parts of the tooth that preserved DNA. However, a DNA yield of 0.0302 ng/µl was achieved from a completely dissolved liquid sample in nitric acid. The DNA amounts obtained from the 24-hour group ranged from 0.244 ng/µl to 0.0260 ng/µl.

The sample exposed to nitric acid for 8 h, which yielded 4.5 ng/µl DNA, was subjected to identification. The results showed alleles from full STR profile (24 STR), achieving a 100% success rate.

The sample exposed to nitric acid for 24 h, which yielded 0.244 ng/µl DNA, was subjected to identification. The results showed alleles from 17 out of 24 STR loci, achieving a 70.83% success rate. Although not all alleles from the 24 STR loci were obtained and the profile was fragmented in 24 h, previous generation identification kits that used 16 STR loci were known to provide a 99.999% identification rate [10]. Thus, it is concluded that the DNA profile with alleles from 17 STR loci can be used to identify the individual. This result indicates that identification is feasible with molar tooth samples exposed to nitric acid for up to 24 h.

### Hydrochloric acid exposure

The effects of hydrochloric acid on dental materials increase in proportion to the duration of exposure. The color of the acidic solution changes in relation to the degree of dissolution of the teeth, with more extensive dissolution resulting in a lighter color. Even when the dental sample is completely dissolved, residues on the outer surface continue to persist.

In the DNA isolation process, 8-hour-exposed samples yielded more successful DNA amounts compared to 24-hour-exposed samples. For the 8-hour exposure, DNA quantities ranged from 0.0426 ng/µl to 7.92 ng/µl. In the 24-hour samples, DNA quantities ranged from 0.0301 ng/µl to 0.171 ng/µl, with one sample failing to yield any detectable DNA. Despite incomplete dissolution of blood and tissue residues on the tooth surfaces in the 24-hour samples, one sample that underwent significant morphological changes became completely liquid. Even from this liquid sample, 0.0398 ng/µl of DNA was recovered.

For identification purposes, a DNA sample with a concentration of 3.15 ng/µl, treated with hydrochloric acid for 8 h, was analyzed. The resulting data yielded alleles from a complete STR profile (24 STR loci), achieving a 100% success rate. Additionally, a DNA sample (Sample 1) with a concentration of 0.171 ng/µl, treated with hydrochloric acid for 24 h, resulted in successful allele recovery from 18 out of 24 STR loci, yielding a 75% success rate. A second DNA sample (Sample 2) with a concentration of 0.154 ng/µl, also treated with hydrochloric acid for 24 h, produced alleles from 13 out of 24 STR loci, resulting in a 54.16% success rate. These findings demonstrate that allele recovery decreases as DNA concentration diminishes. Nevertheless, identification was still achievable in the sample treated with hydrochloric acid for 24 h. Although not all alleles from the 24 STR loci were recovered and the profile was fragmented after 24 h, prior studies using identification kits based on 16 STR loci have reported a 99.999% identification success rate [10]. Therefore, it is concluded that a DNA profile with alleles from 18 STR loci can be utilized for individual identification. This outcome suggests that identification remains feasible for molar tooth samples exposed to hydrochloric acid for up to 24 h.

### Sulfuric acid exposure

In samples exposed to sulfuric acid, no morphological changes were observed after 8 h, while 24-hour samples showed slight erosion and a darker solution color. In 120-hour samples, blood and tissue residues were completely dissolved, resulting in a dark, opaque solution. White residues were noted on the tooth surfaces at each stage, indicating a general interaction effect.

In the DNA isolation process, 8-hour-exposed samples provided more successful DNA amounts compared to 24-hour and 120-hour samples. DNA quantities ranged from 0.0233 ng/µl to 7.65 ng/µl in the 8-hour samples, from 0.0210 ng/µl to 0.255 ng/µl in the 24-hour samples, and from 0.0318 ng/µl to 0.0456 ng/µl in the 120-hour samples. Average DNA recoveries were 1.870 ng/µl for 8-hour samples, 0.0828 ng/µl for 24-hour samples, and 0.0387 ng/µl for 120-hour samples.

For identification purposes, DNA samples with concentrations of 7.65 ng/µl, 0.255 and 0.160 ng/µl, treated with sulfuric acid for 8 h and 24 h respectively, were analyzed. From the sample with a concentration of 7.65 ng/µl treated for 8 h was subjected to identification. The results showed alleles from full STR profile (24 STR), achieving a 100% success rate. Additionally, a DNA sample (Sample 1) with a concentration of 0.160 ng/µl, treated with sulfuric acid for 24 h, produced alleles from 12 out of 24 STR loci, resulting in a 50% success rate. A second DNA sample (Sample 2) with a concentration of 0.255 ng/µl, also treated with sulfuric acid for 24 h, resulted in successful allele recovery from 20 out of 24 STR loci, yielding an 83.3% success rate. Finally, although not enough DNA for identification, a DNA sample with a concentration of 0.0456 ng/µl treated with sulfuric acid for 120 h produced alleles from 8 of the 24 STR loci, resulting in a 30% success rate as a partial profile.

Among the acids evaluated, both nitric acid and hydrochloric acid induced the most significant morphological changes in dental samples. Of the two, nitric acid was found to be the most effective in eliminating residues from tooth surfaces. Although sulfuric acid caused fewer morphological changes, it resulted in the lowest DNA recovery across all groups. Notwithstanding the greater damage caused by sulfuric acid, allele recovery was more successful in samples treated with this acid.

When similar studies in the literature are examined: In a study by Raj et al. the morphological changes induced by nitric acid, hydrochloric acid, and sulfuric acid in dental samples were examined. Like our study, the acids were used at the same concentrations7. In their study, complete dissolution of the teeth occurred after 8 h of exposure to 37% hydrochloric acid. In contrast, our study did not observe complete dissolution after 8 h of exposure. In experiments with 65% nitric acid, the dissolution process was completed within 8 h, which does not align with the results of our study. For experiments conducted with 96% sulfuric acid, a whitish appearance and dissolution in the enamel layer were observed after 3 h, but the tooth morphology remained largely intact even after 8 h. This finding is consistent with our results, where sulfuric acid did not cause significant changes to the tooth surface after 8 h, but over time, it led to the formation of a whitish tissue on the surface, causing dissolution in the enamel [9].

In a study conducted by Turner et al., morphological changes were examined in human teeth soaked in acid. The commercial acids used included 37% hydrochloric acid (HCl), 65% nitric acid (HNO3), and 96% sulfuric acid (H2SO4). This study utilized six anterior canine human teeth, each individually immersed in separate beakers containing one of the acids and observed at various intervals. The findings revealed that the teeth were completely dissolved in 37% hydrochloric acid and 65% nitric acid within 48 h. In contrast, teeth immersed in 96% sulfuric acid did not dissolve completely but instead formed a white precipitate [11]. In the present study, sulfuric acid was determined to be less effective at tooth dissolution. However, due to the inclusion of DNA analysis, it was determined that although sulfuric acid has a lower dissolution capacity, it causes significant DNA damage and DNA quantities are very low, but despite this, identification can be made from samples exposed to sulfuric acid for up to 24 h, and only a partial profile can be obtained from samples exposed for 120 h.

In a study conducted by Snedeker et al., the effect of immersing partial human remains (including whole heads, forearms, and hands) in five different household products—bleach, Rid-X^®^ septic treatment, lye drains opener, sulfuric acid drain opener, and muriatic acid (hydrochloric acid) pool cleaner—were investigated. The researchers assessed the impact of each chemical on visual changes, DNA recovery, and the potential for successful human identification through traditional STR or mitochondrial DNA analyses. Human remains exposed to bleach, Rid-X^®^, and lye generated full STR profiles after 4 weeks of exposure. Sulfuric acid shortened this timeframe to 3 weeks, while hydrochloric acid, the most damaging chemical, restricted full STR profile recovery to just the first 3 days of exposure. In sulfuric acid, tooth samples remained intact until the 5th day, after which they dissolved completely. On day 5, DNA recovery was determined to be 0.2 ng/µl, and identification was considered possible; however, DNA extraction became impossible after this point. Similarly, tooth samples exposed to hydrochloric acid remained intact until day 1, after which they dissolved completely. On day 1, DNA recovery was 2 ng/µl, and identification was deemed possible; however, DNA extraction was not possible after this day [12]. Consistent with these findings, our study demonstrated that DNA profiles could be obtained, and identification was possible from samples exposed to hydrochloric and sulfuric acid for up to 24 h.

Robino et al. conducted DNA typing to identify soft (muscle) and hard (bone and teeth) tissues from pig samples immersed in strong acids (hydrochloric, nitric, and sulfuric acids) or acid mixtures (aqua regia). The samples were subjected to various immersion times, ranging from 2 to 6 h for soft tissues and 2 to 28 days for hard tissues. Soft tissue samples (*n* = 4 per acid) consisted of pork meat specimens (600–800 g) including skin, muscle, fascia, and connective tissue. For hard tissues, sections of adult pig femurs (*n* = 4 per acid) were used, obtained by cross sectioning each bone at the mid-diaphysis. DNA recovery results indicated that compact bone tissue samples could be processed for DNA extraction regardless of immersion time or acid type (e.g., after 2, 7, and 28 days for sulfuric acid; 2 and 7 days for nitric acid; and 2 days for hydrochloric acid and aqua regia). DNA yields were generally high, with 30.6 ng/µl recovered from bone samples treated with sulfuric acid, 4.5 ng/µl from samples treated with nitric acid, and 0.9 ng/µl from samples treated with hydrochloric acid. Complete DNA profiles were obtained for all bone samples using DNA extraction by spin-column methods following pulverization, along with a kit containing 12 pig-specific STR panels [13]. In the present study, DNA recovery reached concentrations of up to 16 ng, consistent with the findings of Robino et al. However, in parallel to Robino et al. using porcine femur bone exposed to acids for up to 2 days and obtaining a complete profile at 13 STRs in pigs, this study focused on human teeth exposed to acids for up to 24–120 h. The results showed that successful DNA isolation and full profile recovery can be achieved from teeth exposed to strong acids for up to 24 h. It is important to note that the femur samples used by Robino et al. were immersed in acid in large sections, likely minimizing degradation compared to the single, isolated teeth used in this study.

In a study by Damascena et al., the effects of various chemical agents on dental samples were evaluated in terms of identification and the impact of acids on DNA. The dental samples were exposed to 37% hydrochloric acid, 10% formaldehyde, and 2.5% sodium hypochlorite for 4 days. Complete dissolution of the teeth was observed because of hydrochloric acid exposure. This complete dissolution rendered DNA recovery impossible, indicating that hydrochloric acid effectively dissolved dental tissue and caused DNA degradation. In our study, the maximum exposure time was 24 h, with only two samples exposed to sulfuric acid for up to 120 h. Therefore, complete dissolution was not observed in all samples. However, like the findings of the study, there were two samples that completely turned into liquid after 24 h of exposure to hydrochloric acid and DNA could be recovered from them [14].

## In conclusion

Morphological results demonstrate that nitric acid is the most effective acid in causing morphological changes, as it visibly and thoroughly destroys all organic structures in and around the tooth. Hydrochloric acid, while it erodes the tooth structure, does not completely dissolve the materials on the surface. Sulfuric acid, although it does not visibly erode the tooth surface, creates a creamy outer layer with increased exposure, which is an important effect to note.

In the DNA isolation procedures conducted in this study, DNA was successfully recovered from 51 out of 52 samples. The use of the QIAGEN QIAamp DNA Investigator Kit was effective for DNA recovery. Despite the extensive morphological damage caused by nitric acid, it was found to yield higher DNA recovery results. The most successful DNA recovery was observed in samples exposed to 8 h of nitric acid, with an average of 4.339 ng/µl. The group with the lowest DNA amount was from samples exposed to sulfuric acid for 120 h, with an average of 0.0387 ng/µl.

DNA recovered from molars was sufficient for identification purposes, while incisors did not provide adequate DNA for identification. These findings underscore the superior efficiency of molars in DNA recovery. Based on DNA profiling, it was concluded that full identification could be achieved from molars exposed to nitric acid, hydrochloric acid, and sulfuric acid for up to 24 h. A complete DNA profile was successfully obtained with 100% accuracy after 8 h of exposure to nitric acid, hydrochloric acid, and sulfuric acid.

For molars exposed to acids for 24 h, alleles were recovered from 20 out of 24 STR loci (83.3% success) in sulfuric acid, 18 out of 24 STR loci (75% success) in hydrochloric acid, and 17 out of 24 STR loci (70.83% success) in nitric acid. While a full DNA profile was not obtained, it is well-established that identification can be achieved with a success rate of 99.999% using as few as 13–16 STR loci.

After 120 h of exposure to sulfuric acid, it was concluded that a partial DNA profile could still be recovered from molars.

Additionally, it was determined that partial DNA profiles could still be recovered from samples that had undergone complete dissolution due to acid exposure, although full identification might not always be feasible. In all acid-soaked samples with partial profiles, alleles were exclusively detected at mini-STR loci (< 150 bp). Given that the GlobalFiler™ PCR Amplification Kit includes 10 mini-STRs, it was concluded that incorporating additional mini-STRs could enhance identification in such challenging cases, achieving up to 99.99% accuracy. Alternatively, more robust identification outcomes could be obtained by utilizing Identification SNP panels.

## Electronic supplementary material

Below is the link to the electronic supplementary material.

Supplementary Material 1

## Data Availability

Not applicable.
