# Supplementary material for: Determination of DNA recovery from human teeth exposed to various acids
Source: Int J Legal Med. 2025 Feb 18;139(4):1453–63. doi: 10.1007/s00414-025-03445-x (PMC12170752; doi:10.1007/s00414-025-03445-x)
Supplement: Supplementary file 1 — Supplementary Material 1 [file 414_2025_3445_MOESM1_ESM.docx]

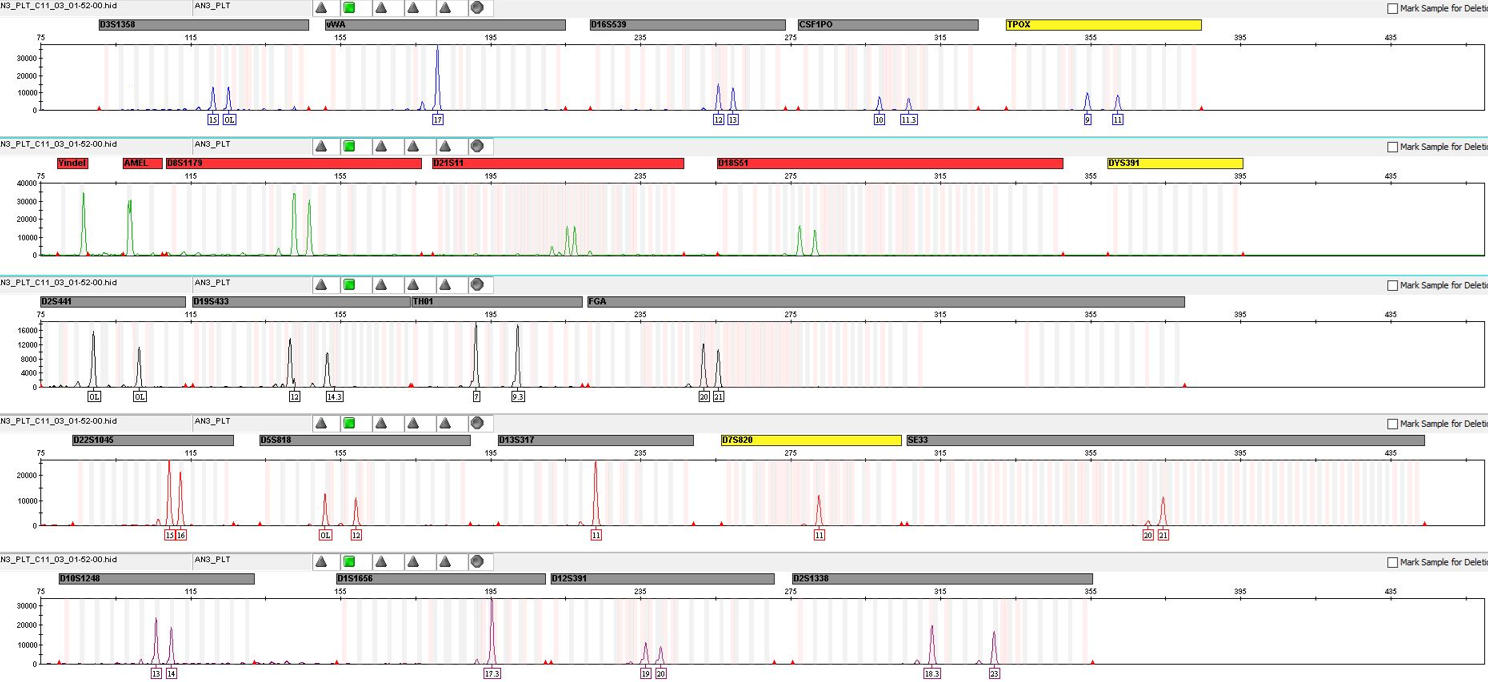


Electropherogram of Samples Exposed to Nitric Acid for 8 Hours


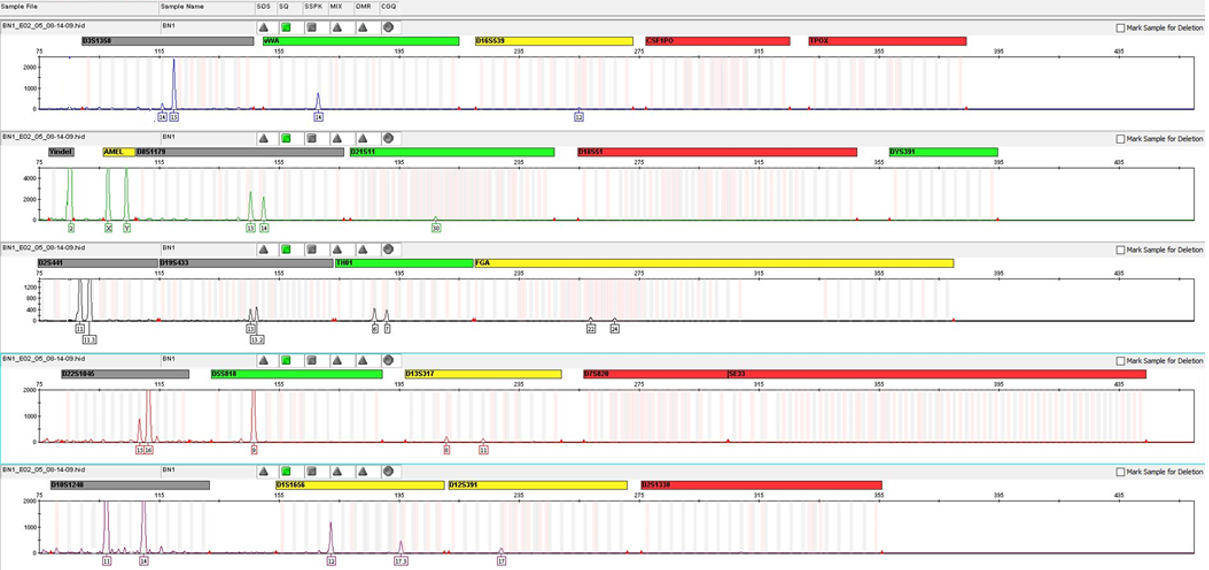


Electropherogram of Samples Exposed to Nitric Acid for 24 Hours


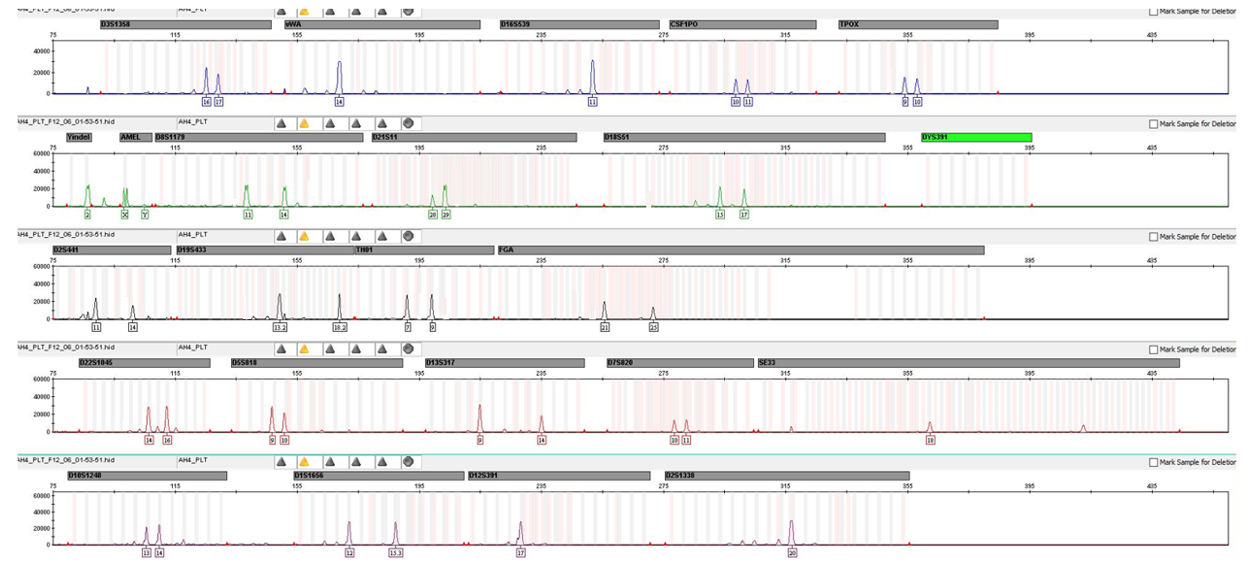


Electropherogram of Samples Exposed to Hydrochloric Acid for 8 Hours


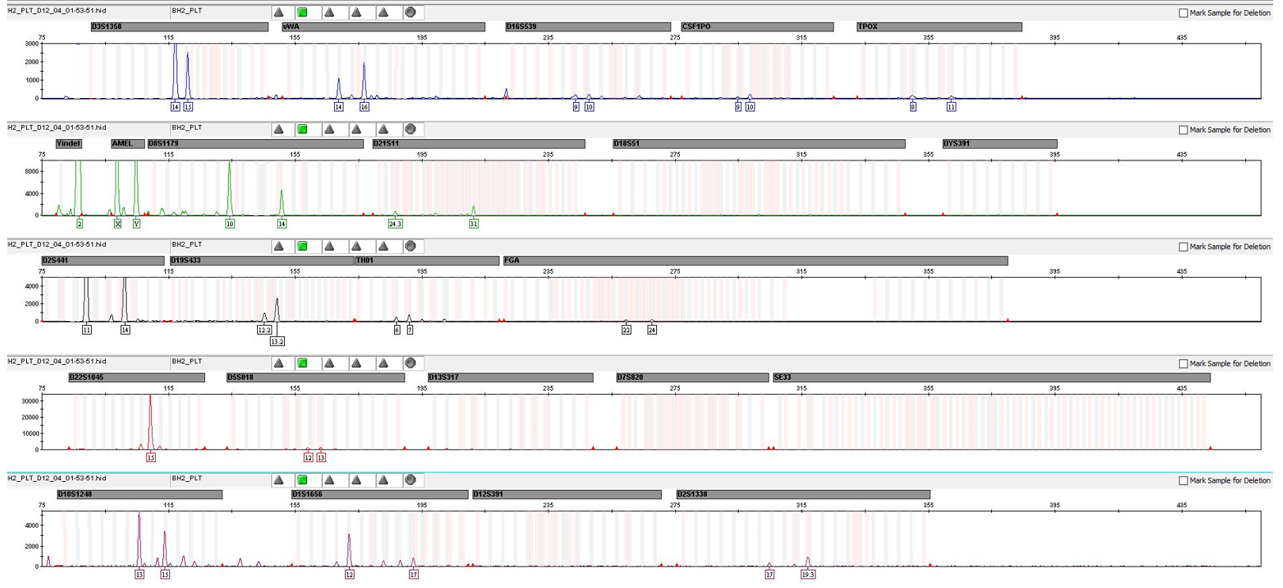


Electropherogram of Samples Exposed to Hydrochloric Acid for 24 Hours (Sample 1)


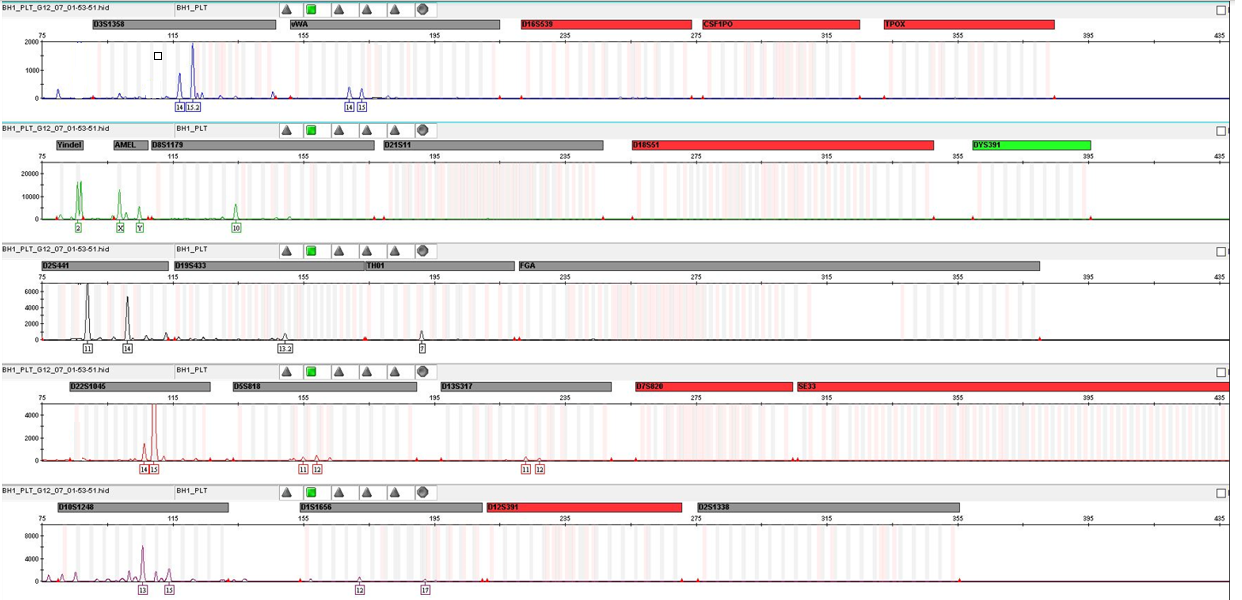


Electropherogram of Samples Exposed to Hydrochloric Acid for 24 Hours (Sample 2)


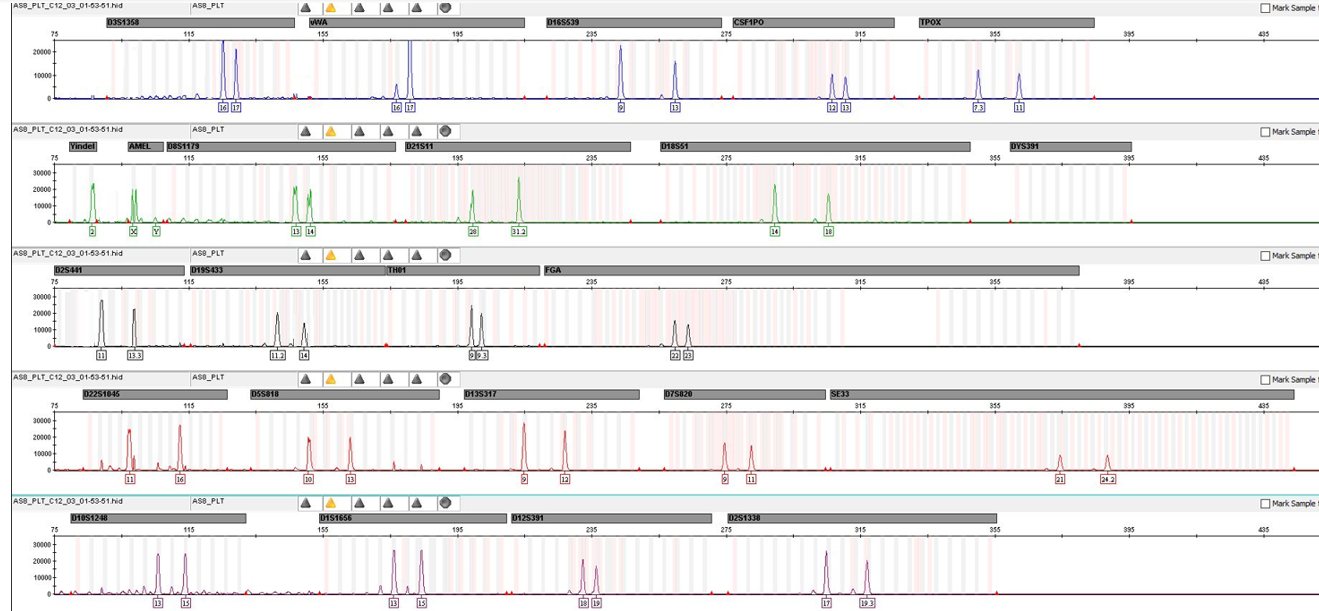


Electropherogram of Samples Exposed to Sulfuric Acid for 8 Hours


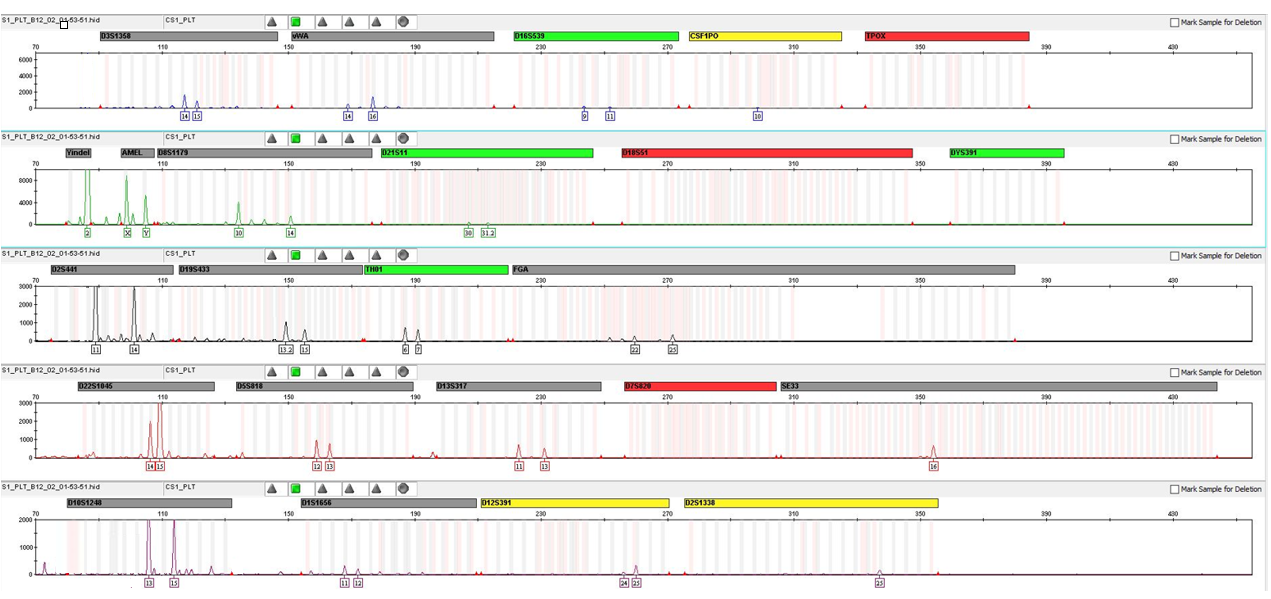


Electropherogram of Samples Exposed to Sulfuric Acid for 24 Hours (Sample 1)


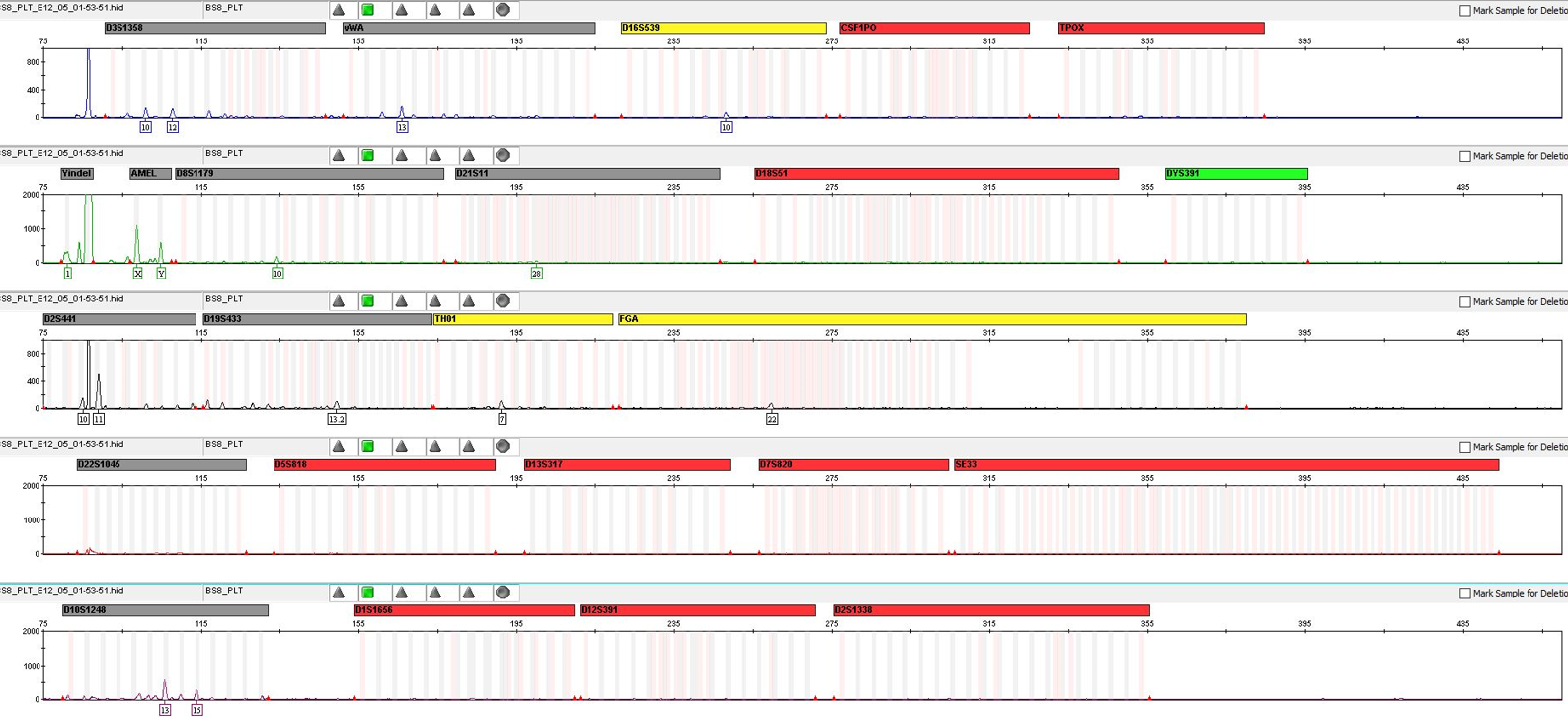


Electropherogram of Samples Exposed to Sulfuric Acid for 24 Hours (Sample 2)


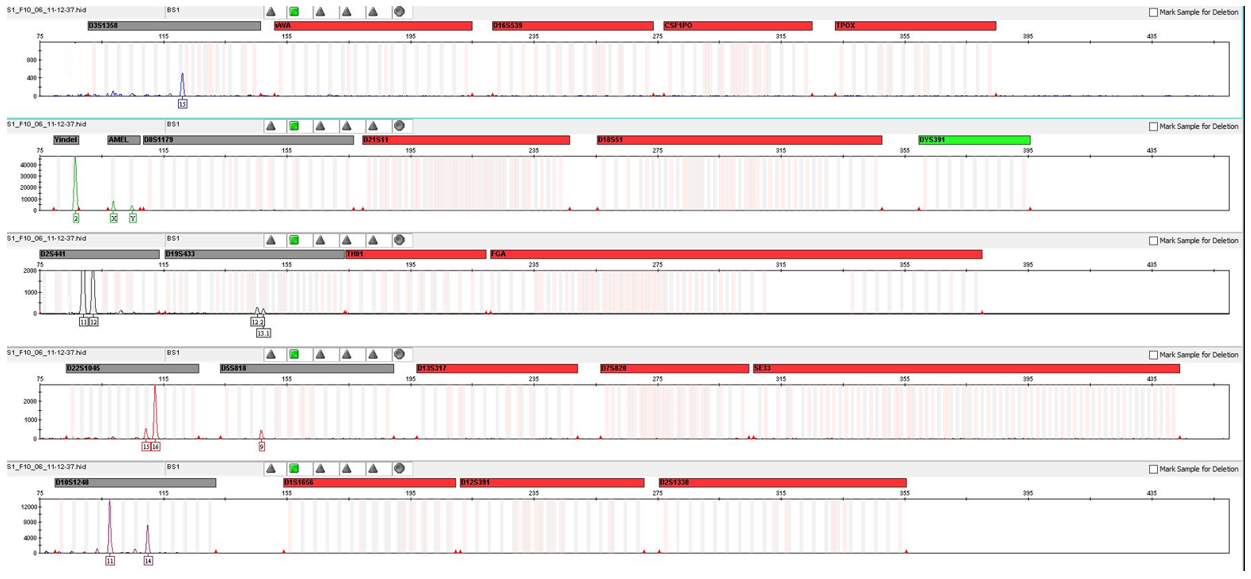


Electropherogram of Samples Exposed to Sulfuric Acid for 120 Hours
